# A Standard for the Measurement of the *p*H of Blood and Other Physiological Media

**DOI:** 10.6028/jres.065A.031

**Published:** 1961-06-01

**Authors:** Vincent E. Bower, Maya Paabo, Roger G. Bates

## Abstract

A buffer solution containing potassium dihydrogen phosphate (0.008695 molal) and disodium hydrogen phosphate (0.03043 molal) is proposed as a *p*H standard for the physiologically important range, *p*H 7 to 8. The proposed standard solution is prepared by dissolving 1.179 g (air weight) of potassium dihydrogen phosphate and 4.303 g (air weight) of disodium hydrogen phosphate in ammonia-free water and diluting to 1 liter at 25 °C. The ionic strength is 0.1.

Standard *p*H values (*p*H_s_) were assigned to this reference solution at temperatures from 0 to 50 °C by means of emf measurements of hydrogen-silver chloride cells without liquid junction. The activity coefficient of chloride ion, upon which the assignment of *p*H_s_ depends, was evaluated by means of a recently proposed convention. By this means, standard values precise to ± 0.001 unit could be derived from the emf data. At 25 °C *p*H_s_ is 7.414, and at 38 °C it is 7.382. The operational consistency of these standard values with those for the 0.025-*m* equimolal phosphate buffer (one of the NBS primary standards) was demonstrated.

## 1. Introduction

The acid-base behavior of blood and other physiological fluids has been widely studied in recent years in medical and biological laboratories in an attempt to discover the relationships that exist among physiological functions, pathological conditions, and *p*H. Many of these fluids are well buffered, and the detection of any systematic variation of *p*H with physiological condition would therefore be expected to require precise measuring equipment. Such equipment is readily available commercially in the form of the newer highly-sensitive *p*H meters with a glass electrode.

Experience has shown that, with the exercise of care, the investigator or clinician can obtain glass-electrode *p*H values, for reduced blood and other body fluids, that agree within ±0.01 unit with *p*H numbers obtained by means of the hydrogen electrode [1].^1^ The high degree of stability in these biological systems and in the measuring apparatus suggests that blood *p*H measurements with a precision of 0.005 unit (or even 0.001 unit) could profitably be made. Meaningful comparisons of highly precise results of different laboratories could then be made, provided that a suitable reference standard were available.

Unfortunately, useful comparisons of *p*H data, quoted to 0.001 unit, from different sources have in general been impossible. The difficulty can be attributed to the fact that in all of the major conventions defining *p*H [2], a nonthermodynamic convention concerning single-ion activity coefficients is by necessity adopted. At the present time no single convention for defining an ionic activity is widely accepted. Although the conventions used heretofore to estimate single-ion activity coefficients are “reasonable” and yield *p*H numbers consistent with each other to within 0.01 unit, they all lack the exactness required for assignment of values to 0.001 unit.

Bates and Guggenheim [3], in a recent report to the International Union of Pure and Applied Chemistry, have proposed a very simple and precise way of defining the single-ion activity for the establishment of *p*H standards. By means of the convention they have set forth, *p*H values precise to within 0.001 unit may be assigned to selected standards in a restricted sector of the *p*H range.

Errors due to the faulty response of the glass electrode are minimized if the *p*H of the standard is close to that of the unknown. The availability of a precise *p*H standard in the physiological range would assist materially in the exact comparison of *p*H measurements made on biological systems in different laboratories.

It is the purpose of this paper to propose, as a physiological *p*H standard, a phosphate buffer mixture with a *p*H of about 7.4 and to assign *p*H values to this standard. The proposed standard has the following composition: KH_2_PO_4_(0.008695 molal), Na_2_HPO_4_(0.03043 molal).

## 2. Method of Defining *p*H_s_

In order to avoid the theoretical and practical difficulties inherent in the estimation of liquid junction potentials, standard *p*H values have been based upon measurements of cells without liquid junction. In this work, hydrogen and silver-silver chloride electrodes were used, measurements being made of the electromotive force of the cell:

$$\begin{array}{l} \text{Pt};H_{2}(g,1\;\text{atm}),\;{\text{KH}}_{2}{\text{PO}}_{4}(0.008695\;\text{molal}),\\ {\text{Na}}_{2}{\text{HPO}}_{4}(0.03043\;\text{molal}),\;{\text{Cl}}^{-},\text{AgCl};\text{Ag}. \end{array}$$ (A)

The electromotive force, *E*, of cell (A) is related to the standard electrode potential (*E*°) of the silver-silver-chloride electrode [4] and to the activities of the hydrogen and chloride ions in the solution by the relation

$$E=E{}^{\circ}-\frac{RT}{F}\ln(a_{H}a_{\text{Cl}}),$$ (1)

where ***a*** is the activity (molal basis) of the ionic species designated by the subscript. Rearrangement, conversion to decadic logarithms, and substitution of *m*_Cl_γ_Cl_ for *a*_Cl_ yields a thermodynamic acidity function, −log(γ_H_γ_Cl_*m*_H_):

$$-\log(\gamma_{H}\gamma_{\text{Cl}}m_{H})=(E-E{}^{\circ})F/(2.3026RT)+\log m_{\text{Cl}}.$$ (2)

In eq (2)*m* is the molality and γ is the activity coefficient, on the molal scale, of the ions designated by the subscripts.

For satisfactory reproducibility and constancy of the electromotive force, a finite amount of chloride must be present in the buffer solution. Nevertheless, it is the acidity function of the chloride-free buffer solution that is desired. The value of −log(γ_H_γ_Cl_*m*_H_) was therefore measured at each of three low chloride concentrations, namely 0.005 *m*, 0.010 *m*, and 0.015 *m* and −log(γ_H_γ_Cl_*m*_H_)°, the limit of −log(γ_H_γ_Cl_*m*_H_) as *m*_Cl_ approaches zero, was obtained by extrapolation.

If the standard *p*H (denoted *p*H_s_) is defined formally as −log ***a***_H_ or −log *m*_H_γ_H_, then

$$pH_{s}=-\log{\left(\gamma_{H}\gamma_{\text{Cl}}m_{H}\right)}^{{}^{\circ}}+\log\gamma_{\text{Cl}}^{{}^{\circ}}.$$ (3)

It will be observed that, according to eq (2), −log(γ_H_γ_Cl_*m*_H_) is a measurable quantity. On the contrary, $\gamma_{\text{Cl}}^{{}^{\circ}}$ in eq (3) is the activity coefficient of a single ion, a quantity that is not measurable. To obtain *p*H_s_, therefore, some assumption must be made, or convention adopted, to evaluate $\gamma_{\text{Cl}}^{{}^{\circ}}$.

For the purposes of assigning standard *p*H values to the standard buffers already proposed by the National Bureau of Standards, various assumptions have been used to estimate $\gamma_{\text{Cl}}^{{}^{\circ}}$ [5,6,7]. If the ionic strength does not exceed 0.1, the numerical values of $\gamma_{C}^{{}^{\circ}}$ (obtained from the above assumptions) can all be closely represented by the following form of the Debye-Hückel equation:

$$-\log\gamma_{\text{Cl}}^{{}^{\circ}}=\frac{A\sqrt{\mu }}{1+Ba_{i}\sqrt{\mu }},$$ (4)

where *μ* is the ionic strength, *a_i_* is an adjustable “ion-size parameter”, and *A* and *B* are constants dependent upon temperature and solvent.^2^ In the last analysis, therefore, the differences among the conventions themselves can be expressed as differences in *a_i_*. Fortunately, all “reasonable” conventions for the definition of $\gamma_{\text{Cl}}^{{}^{\circ}}$ lead to substantially equivalent values for the *p*H_s_ of the standard phosphate buffer (*μ* = 0.1) [10]. All of these values fall within ±0.01 unit of the assigned NBS standard values for this buffer solution. The agreement at lower ionic strengths is even more satisfactory.

Since there is really no apparent basis for choice among these assumptions, any one of them may be selected to represent $\gamma_{\text{Cl}}^{{}^{\circ}}$. Bates and Guggenheim have proposed, in their report to the Analytical Chemistry and Physical Chemistry Sections of the International Union of Pure and Applied Chemistry, a value of B*a_i_* = 1.5 mole^−½^ kg^½^ [3]. The *p*H values given in the present work are based upon eqs (3) and (4) and this convention of Bates and Guggenheim.

## 3. Experimental Procedures and Results

The cells used for the measurements have been described in detail in a previous article [6]. Briefly, each cell consists of two electrode compartments and a chamber in which incoming hydrogen is saturated with the vapor over the buffer solution. The chamber terminates in a tube which leads to the jet in the hydrogen electrode compartment of the cell. Hydrogen gas escapes from the top of the hydrogen electrode compartment. The silver-silver chloride electrode compartment is connected to the hydrogen electrode compartment by a broad tube filled with the cell solution.

The standard buffer solution selected was 0.008695 molal in potassium dihydrogen phosphate (molecular weight, 136.092) and 0.03043 molal in disodium hydrogen phosphate (molecular weight, 141.982).^3^ These proportions were chosen with the intention of producing a solution of *p*H about 7.4 at 25 °C and with an ionic strength of 0.1.

The solvent used in this study was ammonia-free distilled water of conductivity no greater than 0.8×10^−6^ ohm^−1^ cm^−1^. The phosphate salts were specimens of NBS Standard Sample 186Ib (KH_2_PO_4_) and 186IIb (Na_2_HPO_4_), dried for an hour in a oven at 110 °C and used without further treatment. The potassium chloride was taken from a highly purified sample whose manner of preparation and purification has been described elsewhere [11].

Twenty cells containing the phosphate buffer with low concentrations of chloride (0.005 *m*, 0.010 *m*, or 0.015 *m*) were made up. The emf of 13 of these cells was measured over the temperature range 0 to 50 °C; five cells were studied over the range, 25 to 50°, one cell over the range 0 to 40°, and one cell at only three temperatures.

After the emf had been corrected to the standard partial pressure of hydrogen (760 mm), the acidity function −log(γ_H_γ_Cl_*m*_H_) for each cell was calculated by means of eq (2). Values of −log (γ_H_γ_Cl_*m*_H_) are listed in table 1. For these calculations, *R* was taken as 8.3147 j mole^−1^ deg^−1^ and *F* as 96,495.4 coul equiv^−1^ [12].

## 4. Assignment of *p*H_s_ Values

**Figure 1:**
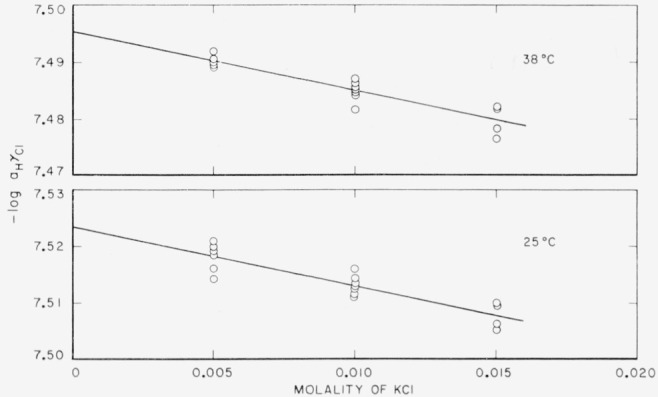
−log (γ_H_γ_Cl_*m*_H_) of a solution 0.008695*m* in *KH_2_PO_4_* and 0.03043 *m* in *Na_2_HPO_4_* as a function of molality of added chloride.

**Figure 2:**
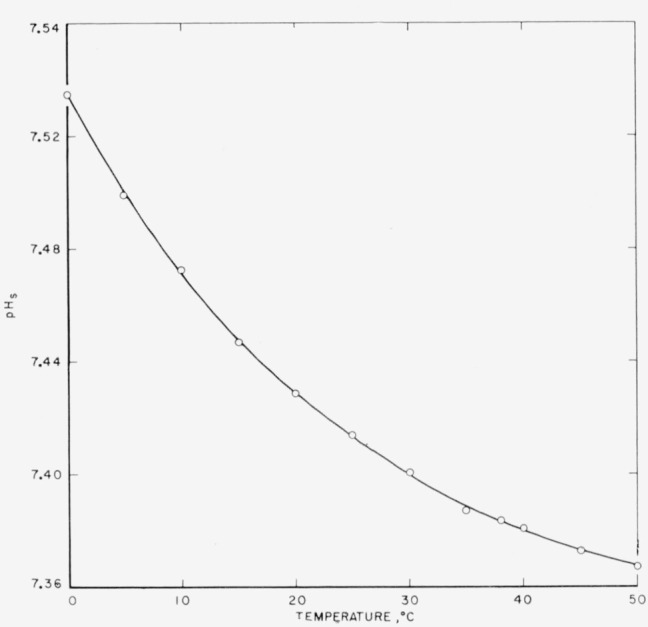
*p*H_s_ of a solution 0.008695*m* in *KH_2_PO_4_* and 0.03043 *m* in *Na_2_HPO_4_* as a function of temperature.

The −log(γ_H_γ_Cl_*m*_H_) was found to be a linear function of *m*_Cl_, and the limiting value, −log(γ_H_γ_Cl_*m*_H_)° (at *m*_Cl_ = 0), was evaluated by the method of least squares. Figure 1 shows the array of −log(***a***_H_γ_Cl_) or −log(γ_H_γ_Cl_*m*_H_) values at two temperatures. Values of −log(γ_H_γ_Cl_*m*_H_)° and the standard deviation (*σ_i_*) of the intercept are given in table 2. The quantities −logγ°_Cl_ computed from eq (4) are also listed. The standard *p*H_s_ values were derived from −log(γ_H_γ_Cl_*m*_H_)° by eq (3). They are listed in the last column of table 2 and are represented graphically as a function of temperature in figure 2.

The standard solution proposed here has an ionic strength of 0.1 and a buffer ratio of 3.5. The difference in *p*H resulting from small variations in the concentration of one or both phosphates is given with sufficient accuracy by the following expression, based on the mass law:

$$\begin{array}{l} pH_{s}-pH_{x}=0.544-\log\frac{m_{{\text{HPO}}_{4}^{--}}}{m_{H_{2}{\text{PO}}_{4}^{-}}}\\ +3\{\log\gamma_{\text{Cl}}^{{}^{\circ}}(s)-\log\gamma_{\text{Cl}}^{{}^{\circ}}(x)\}, \end{array}$$ (5)

where x designates the solution of buffer ratio slightly different from 3.5, and/or ionic strength slightly different from 0.1, and where s designates the standard solution proposed here. Also assumed in the derivation of eq 5 was the approximation

$$\gamma_{{\text{HPO}}_{4}^{--}}={\left(\gamma_{\text{Cl}-}^{{}^{\circ}}\right)}^{4}={\left(\gamma_{H_{2}{\text{PO}}_{4}^{-}}\right)}^{4}.$$

It should be noted that neither of these phosphate salts is a strong enough acid or base to react appreciably with water. Hydrolysis corrections are therefore unnecessary, and the ratio of the concentrations of the phosphate ions is the same as the stoichiometric ratio of the molalities of the two salts. The first term within the braces in eq (5) is given in table 2. The second quantity inside the braces may be calculated from eq (4) with *B*a*_i_*=1.5 mole^−½^kg^½^. For buffer solutions composed of potassium hydrogen phosphate (*m*_1_) and disodium hydrogen phosphate (*m*_2_), *μ* = *m*_1_+3*m*_2_.

The “experimental” values of *p*H_s_ listed in table 2 were smoothed with respect to the temperature (*T*) in °K by the method of least squares, with the following result:

$$pH_{s}=\frac{1592.07}{T}-2.3392+0.014798T.$$ (6)

Recommended values of *p*H_s_, calculated at specified temperatures from eq (6), are given in table 3.

## 5. Internal Consistency of the Standard *p*H Scale

It was considered desirable to test the operational consistency of the *p*H*_s_* values defined in this paper with those defined some years ago by Bates and Acree for the equimolal 0.025 *m* phosphate buffer (0.025 *m* KH_2_PO_4_, 0.025 *m* Na_2_HPO_4_) [6]. To this end, emf measurements were made with a symmetric cell [13] consisting of two hydrogen electrode compartments connected by a bridge of saturated potassium chloride.

The standard equimolal phosphate buffer solution was placed in one of the hydrogen electrode compartments and the solution of buffer ratio 3.5 in the other. The emf between the two hydrogen electrodes yields the operational *p*H difference (Δ*p*H), which can then be compared with the difference of assigned *p*H_s_ (Δ*p*H_s_). The assignment of *p*H_s_ is made by eq (3), where log $\gamma_{\text{Cl}}^{0}$ is defined by the Bates-Guggenheim convention (and therefore constant for a given value of the ionic strength).^4^ The difference in *p*H_s_ is therefore equal to the difference between the values of −log(γ_H_γ_Cl_*m*_H_)° for the two solutions, namely 7.523–6.972 or 0.551 at 25 °C and 7.496–6.952 or 0.544 at 38 °C.

Measurements of the symmetrical cell with liquid junction were made at 25 and 38 °C. The experimental values of Δ*p*H at the two temperatures were, respectively, 0.550 and 0.546 *p*H unit. These values are in good agreement with the values of Δ*p*H_s_ given above, which were, of course, derived from the emf of cells without liquid junction.

The internal consistency of the practical scale to a few thousandths of a unit in the physiological range therefore seems to have been demonstrated. By a similar series of measurements at 25 °C, it has already been shown that the standard phthalate (*p*H 4.006), phosphate (*p*H 6.863), and borax (*p*H 9.183) buffer solutions are consistent among themselves to about ±0.003 unit [14]. In the authors’ opinion, these intercomparisons constitute an experimental justification for the assignment of a third decimal to the *p*H_s_ for the primary standards of intermediate *p*H.

## Figures and Tables

**Table 1:** Electromotive force of cell (A) containing the phosphate buffer solution: 0.008695 *m* kh_2_po_4_, 0.03043 *m* Na_2_HPO_4_ with three molalities of added KCl

Temperature °C												
KCl, molality	0	5	10	15	20	25	30	35	38	40	45	50
												
0.005	0.77510	0.78078	0.78631	0.79187	0.79754	0.80324	0.80897	0.81457	0.81800	0.82018	0.82577	0.83149
.005	.77481	.78037	……………	……………	……………	.80308	……………	……………	……………	……………	……………	……………
.005	.77502	.78045	.78607	.79168	.79745	.80322	.80888	.81451	.81796	.82018	.82586	.83145
.005	.77484	.78042	.78612	.79177	.79756	.80296	.80893	.81434	.81791	.82021	.82606	.83180
.005	.77537	.78080	.78650	.79198	.79771	.80336	.80902	.81469	.81810	.82048	……………	……………
.005	.77531	.78063	.78612	.79149	.79722	.80326	.80884	.81450	.81794	.82025	.82578	.83142
.005	……………	……………	……………	……………	……………	.80330	.80884	.81435	.81798	.82025	.82566	.83122
.005	……………	……………	……………	……………	……………	.80322	.80886	.81450	.81798	.82036	.82612	.83151
.010	.75857	.76392	.76912	.77442	.77970	.78512	.79047	.79584	.79916	.80124	.80672	.81222
.010	.75835	.76380	.76899	.77424	.77958	.78508	.79070	.79588	.79909	.80115	.80663	.81200
.010	.75862	.76374	.76903	.77421	.77946	.78506	.79044	.79568	.79903	.80136	.80667	.81188
.010	.75869	.76384	.76915	.77445	.77989	.78506	.79056	.79588	.79911	.80133	.80678	.81224
.010	.75869	.76379	.76920	.77438	.77979	.78500	.79048	.79587	.79906	.80129	.80662	.81222
.010	.75868	.76381	.76922	.77435	.77970	.78517	.79054	.79596	.79921	.80143	.80641	.81235
.010	……………	……………	……………	……………	……………	.78497	.79026	.79563	.79887	.80107	.80659	.81195
.010	……………	……………	……………	……………	……………	.78527	.79048	.79585	.79920	.80135	.80673	.81195
.015	.74873	.75392	.75900	.76406	.76923	.77427	.77966	.78478	.78800	.78993	.79513	.80036
.015	.74859	.75360	.75875	.76383	.76895	.77421	.77949	.78443	.78768	.78966	.79501	.80042
.015	.74895	.75373	.75895	.76394	.76918	.77449	.77947	.78476	.78779	.78999	.79521	.80067
.015	……………	……………	……………	……………	……………	.77446	.77978	.78498	.78803	.79017	.79546	.80049

**Table 2:** *p*H_s_ and −log (γ_H_γ_Cl_*m*_H_)° for the solution 0.008695 *m* in KH_2_PO_4_ and 0.03043 *m* in Na_2_HPO_4_

*t*°C	−log(γ_H_γ_Cl_*m*_H_)°	*σ_i_*	$-\log\gamma_{\text{Cl}}^{{}^{\circ}}$	*p*H*_s_* (experimental)
				
0	7.6400	0.0023	0.1055	7.534
5	7.6052	.0018	.1062	7.499
10	7.5793	.0018	.1070	7.472
15	7.5547	.0017	.1078	7.447
20	7.5374	.0019	.1087	7.429
25	7.5234	.0011	.1095	7.414
30	7.5109	.0011	.1104	7.400
35	7.4985	.0014	.1114	7.387
38	7.4955	.0011	.1121	7.383
40	7.4934	.0013	.1125	7.381
45	7.4863	.0016	.1135	7.373
50	7.4816	.0018	.1146	7.367

**Table 3:** Recommended values of *p*H*_s_* at specified temperatures (values calculated from eq (6))

*t*	*p*H*_s_*
	
0	7.531
5	7.501
10	7.474
15	7.450
20	7.430
25	7.413
30	7.399
35	7.387
38	7.382
40	7.379
45	7.373
50	7.369

## Footnotes

1 Figures in brackets indicate the literature references at the end of this paper

2 Tabulations of *A* and *B* are to be found in references [8] and [9].

3 The density at 25 °C of this solution is 1.0020 g/ml. A solution of this composition may be made by dissolving 1.179 g (air weight) KH_2_PO_4_ and 4.303 g (air weight) Na_2_HPO_4_ in water and diluting to 1 liter at 25 °C.

4 The earlier assignment of *p*H*_s_* to the phosphate buffer solution was based on a more complex formula for estimating $\gamma_{\text{Cl}}^{0}$ [6]. The two procedures lead to values of *p*H*_s_* for the equimolal phosphate standard that differ by only 0.003 unit at 25 °C.
